# Engineered extracellular vesicles as therapeutics of degenerative orthopedic diseases

**DOI:** 10.3389/fbioe.2023.1162263

**Published:** 2023-06-09

**Authors:** Junyu Wei, Zixuan Ou, Bide Tong, Zhiwei Liao, Cao Yang

**Affiliations:** Department of Orthopaedics, Union Hospital, Tongji Medical College, Huazhong University of Science and Technology, Wuhan, China

**Keywords:** extracellular vesicles (EVs), native EVs, engineered EVs, degenerative orthopedic diseases (DODs), osteoarthritis (OA), osteoporosis (OP), intervertebral disc degeneration (IDD), osteonecrosis of the femoral head (ONFH)

## Abstract

Degenerative orthopedic diseases, as a global public health problem, have made serious negative impact on patients’ quality of life and socio-economic burden. Traditional treatments, including chemical drugs and surgical treatments, have obvious side effects and unsatisfactory efficacy. Therefore, biological therapy has become the focus of researches on degenerative orthopedic diseases. Extracellular vesicles (EVs), with superior properties of immunoregulatory, growth support, and drug delivery capabilities, have emerged as a new cell-free strategy for the treatment of many diseases, including degenerative orthopedic diseases. An increasing number of studies have shown that EVs can be engineered through cargo loading, surface modification, and chemical synthesis to improve efficiency, specificity, and safety. Herein, a comprehensive overview of recent advances in engineering strategies and applications of engineered EVs as well as related researches in degenerative orthopedic diseases, including osteoarthritis (OA), osteoporosis (OP), intervertebral disc degeneration (IDD) and osteonecrosis of the femoral head (ONFH), is provided. In addition, we analyze the potential and challenges of applying engineered EVs to clinical practice.

## 1 Introduction

Degenerative orthopedic diseases (DODs), referring to gradual destructive changes in the joint, spine, and bone quality, have now become a global public health problem as the elderly population increases (Calvit and Aranda, 1995; Atesok et al., 2017; Pei et al., 2022). Common DODs such as osteoarthritis (OA), intervertebral disc degeneration (IDD), osteoporosis (OP), osteonecrosis (ON), and tendinopathy can cause pain and joint stiffness in patients, resulting in a great impact on the quality of life of patients and a huge economic burden on society (Calvit and Aranda, 1995; Atesok et al., 2017; Xie et al., 2020; Pei et al., 2022; Wu et al., 2022). At present, the treatments of DODs mainly include drug therapy and surgical treatment (Englund et al., 2012; Dowdell et al., 2017; Abramoff and Caldera, 2020). However, drug treatment can lead to obvious adverse reactions and ultimately cannot avoid joint and cartilage degeneration, while surgery is traumatic and expensive, which leads to the limited application of both. In recent years, cell-based therapy has aroused extensive attention owing to its potential for anti-inflammatory, immune regulation, and growth support (Mathis and Hess, 1988; De Bari and Roelofs, 2018; Macías et al., 2020; Krut et al., 2021). However, the clinical application of cell-based therapies remains limited due to the poor survival of implanted cells *in vivo*. It has been reported that the implanted cells play a therapeutic role mainly by secreting extracellular vesicles (EVs) in the form of paracrine (Wysoczynski et al., 2018; Vagnozzi et al., 2020). This has led people to turn their attention to the treatment of DODs with EVs, hoping to replace cell therapy with cell-free therapy.

EVs is a general term for heterogeneous bilayer lipid membrane vesicles, usually 30–2,000 nm in diameter (Kubo, 2018; Shah et al., 2018; Ortiz, 2021). EVs can mediate intercellular communication as carriers of various bioactive signal molecules and have become a hot spot in the study of diseases and tissue repair (Mathieu et al., 2019). EVs are molecules containing a variety of biological activities, including proteins, messenger RNA, microRNA (miRNA/miR), fat, and DNA, and can be divided into two main categories named exosomes (Exos), microvesicles (MVs), and apoptotic bodies (ApoBDs) based on diameter and biogenic pathway (Namee and O’Driscoll, 2018; Marar et al., 2021). Exos are the smallest EVs, ranging from 30 to 150 nm in diameter, produced by multivesicular endosomes and released with the fusion of compartments and plasma membranes (Chuanjiang et al., 2018). MVs are 50–1,000 nm in diameter and can be directly detached from the plasma membrane. Similar to EXOs, MVs may contain nucleic acids, proteins, and lipids, and can transport the above-mentioned signaling molecules to target cells (Ratajczak and Ratajczak, 2020). ApoBDs are the largest known EVs with a diameter of more than 1,000 nm and are formed in the late stage of apoptosis (Shah et al., 2018). To maintain the accuracy of the information, we report the terms used by the authors in their original work to refer to each cited study.

In recent years, many studies have reported the important therapeutic effects of native EVs in DODs, including anti-inflammatory, reducing chondrocyte apoptosis, and promoting tissue repair (Silverman, 1986; Joseph et al., 2016; Lee et al., 2021; Mustonen and Nieminen, 2021). However, several challenges such as insufficient targeting, easy removal, and limited effect have been encountered in the application of native EVs to treat DODs. To overcome these drawbacks, researchers have begun to prepare engineered EVs by nuclear drug loading, membrane modification, or changing the growth environment of donor cells. Engineered EVs not only have the excellent biocompatibility of natural EVs but also have the advantages of controllable drug loading concentration and specific target recognition.

In this review, we first introduce the properties of EVs, this will be followed by a discussion of the application of native EVs in treating DODs. Then we present the methods of engineering EVs including surface modification and content loading in DODs and analyze the prospects and challenges in this exciting field in the future.

## 2 Properties of EVs

EVs are a heterogeneous group of natural particles whose nomenclature, isolation and characterization is described comprehensively in the MISEV2018 position paper from the International Society for Extracellular Vesicles (ISEV) (Théry et al., 2018).

One of the challenges of EVs characterization is the lack of highly specific biomarkers. Although several surface proteins (such as CD9, CD63 and CD81, *etc.*) that are heterogeneously expressed on EVs are generally accepted as markers. However, there are still a few EVs that do not carry any of the above typical markers (He et al., 2019; Tian et al., 2020; Alfaro et al., 2021; Mizenko et al., 2021). Therefore, current characterization techniques based on fluorescent labeling. Nanoflow and total internal reflection fluorescence microscopy (TIRF) tend to label some EVs subgroups while ignoring others. To solve the above difficulties, traditional labeling techniques (such as surface plasmon technique and super resolution microscopy) have also been used to characterize EVs (Srujan et al., 2020; Alfaro et al., 2021). Due to the complexity of exosome source and composition, the difficulties caused by exosome heterogeneity need to be paid attention to and overcome in current studies.

A brief description of EV properties, including biological characteristics, biogenesis and uptake, is provided below. Researchers can modify these properties to produce engineered EVs.

### 2.1 Biological characteristics of EVs

#### 2.1.1 Membrane composition

EVs are extracellular structures composed of lipid bilayers, and their membranes contain proteins, lipids, sugars, *etc.* Due to the different origins of cells, the composition of the vesicle membrane is different, and the therapeutic effect of EVs on diseases also differs. The Exo membrane, which contains more cholesterol than the donor membrane, has been reported to have high levels of phosphatidylserine, sphingomyelin, and glycosphingolipids but low levels of phosphatidylcholine (Xabier OsteikoetxeaBalogh et al., 2015; Skotland et al., 2017). In addition to lipids, the Exo membrane also contains proteins and sugars, which help maintain the Exo membrane structure and facilitate its interaction with target cells. However, according to relevant studies, Exo’s protein components are different from other EVs. It does not contain proteins in the endoplasmic reticulum, but highly expresses tetraspanins that interact with major histocompatibility complexes and integrins, such as CD9, CD81, and CD63 (Kalluri and LeBleu, 2020; Ratajczak and Ratajczak, 2020). These proteins are also considered markers of Exos and play important roles in cell adhesion and membrane fusion. In addition to tetraspanins, there are cell adhesion proteins (ICAM, integrin, lactadherin), intracellular transport proteins (RAB, GTPases, annexins), cell type-specific proteins (MHC-I, MHC-II, APP, PMEL, TCR, FasL, CXCR4), and so on (Guillaume et al, 2018). The surface of Exos also contains a large number of transcription factors, soluble molecules, and growth factors, which enable Exos to participate in a variety of physiological activities in the body and exert a huge regulatory effect on the growth and development of the body (Abels and Breakefield, 2016; Todorova et al., 2017). Notably, the corresponding molecular markers on Exos, MVs, and ApoBDs are different due to the different types of proteins present in them. Selectins, integrin, and CD40 were the main biomarkers for MVs, while caspase3 and histones were the molecular markers for ApoBDs (Todorova et al., 2017). Studies have shown that changing the sugar or protein composition on the membrane of EVs can affect vesicle tropism and physiological properties (D Michiel and Gould, 2019; van Niel et al., 2018). Therefore, modification of cell membrane components is an important way to engineer EVs.

#### 2.1.2 Cargo

The contents of EVs are not static but vary according to cell type, physiological condition, and mode of biogenesis. In summary, EVs can carry cargo such as proteins, lipids, and nucleic acids to transmit signals between cells. The exploration of cargo has been ongoing for decades, and high-resolution density gradient segmentation and direct immunoaffinity analysis have provided a deeper understanding of the contents of EVs (Jeppesen et al., 2019). The lumen of EVs contains a large number of proteins, most of which are related to biogenesis, such as proteins related to the endosomal pathway (ALIX, TSG101), and EV transport (RAB27A, ARF6) (Abels and Breakefield, 2016; Pegtel and Gould, 2019). In addition, there are various enzymes (RNA editing enzymes, lipase, protease), transmembrane proteins (LAMP1. TFR), *etc.* (Todorova et al., 2017). Studies have shown that miRNAs and mRNAs are the main components of EVs, while with the progress of technology, more RNA species such as small nuclear RNAs, Y RNAs, and repetitive element RNAs have been observed (Abels and Breakefield, 2016; Zhang et al., 2021a). These RNAs are on average around 200 nucleotides in length, and smaller RNAs can be up to 4 KB (Abels and Breakefield, 2016; Lakshmi et al., 2021). EVs also contain a variety of DNA, including single-stranded DNA, double-stranded DNA, genomic DNA, and so on (Elzanowska et al., 2021). Related DNA sequencing has also revealed the genome sequence of EVs, but many questions remain regarding DNA, it is not clear which DNA is in organelles and which is bound to the surface, whether DNA secreted by EVs contributes to DNA quality control and whether it is a useful marker for cancer and viral infection. It should be noted that the contents of EVs are not random. Each EV carries specific molecular information, and there is a complex sorting system in the body to determine which molecules can enter the EVs and become the cargo of the EVs. For example, proteins can be sorted into EVs by post-translational modification and mRNAs sorted into EVs often contain 3’UTR fragments (Juan and Fürthauer, 2017; Anand et al., 2019; Wei et al., 2021a). The sorting process of proteins, lipids, DNA, and RNA in the body is complex. Moreover, the research on the sorting mechanism of DNA and RNA is still in the stage of continuous exploration.

### 2.2 Biogenesis of EVs

**FIGURE 1:**
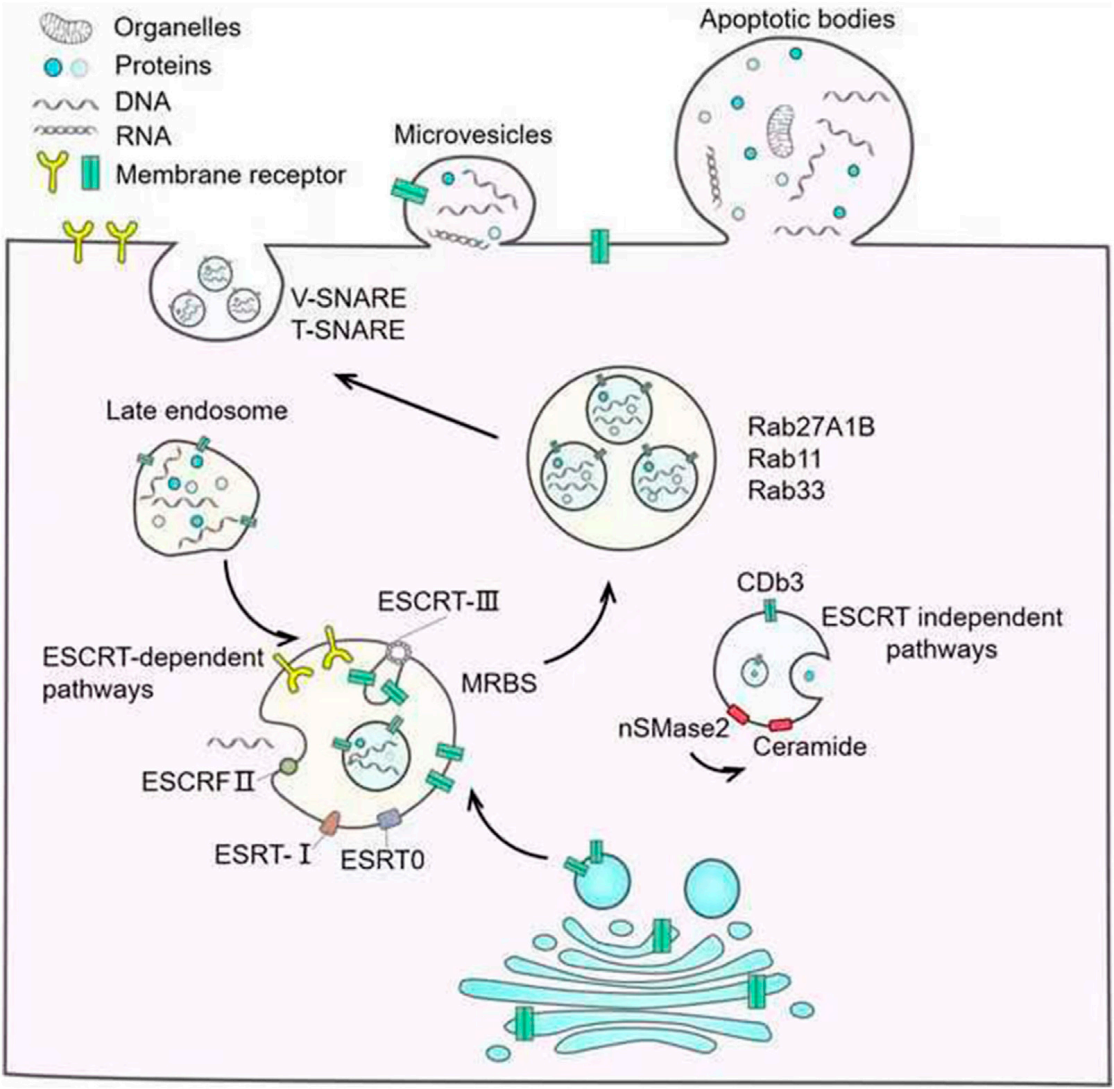
Biogenesis of EVs.

The biogenesis of EVs is complex, and the types of EVs formed by different cells are heterogeneous. In the following, the biogenesis of EVs is briefly summarized according to different types, as is briefly shown in Figure 1.

#### 2.2.1 Exos

At present, the most studied mechanism of Exo generation is the endosome pathway. The forming process of Exos begins with the formation of early endosomes formed by endocytosis on the surface of the cell membrane, and then the early endosomes mature into late endosomes. The late endosomes wrap specific sorted proteins, nucleic acids, and other substances to form multiple luminal vesicles through inward budding, which are the precursors of Exos (Simons and Raposo, 2009). The late endosomes contain multiple ILVs and become multivesicular bodies (MVBs). Subsequently, most MVBs fuse with lysosomes, leading to degradation of the MVB inclusions, while a few MVBs have CD63, lysosomal membrane protein LAMP1, and LAMP2 on their membrane surfaces, which mediate their fusion with the cytoplasmic membrane and release of Exos extracellularly (Fujita et al., 2015). There are two mechanisms for MVB formation, including transport (ESCRT) dependent endoplast sorting complex and ESCRT independent mechanism (Henne et al., 2011; Mashouri et al., 2019). ESCRT is a protein complex located on the cytoplasmic side of endocytosis, whose primary role is to classify specific components into ILVs (Henne et al., 2011). ESCRT contains four complexes including ESCRT-0, I, Ⅱ, Ⅲ and accessory proteins such as VPS4, VTAl, and ALIX (Jimenez et al., 2014). The four complexes have different components and play different roles in the generation of ILVs and MVBs. ESCRT-dependent mechanisms to produce MVBs require 4 steps including cargo aggregation, membrane invagination, cargo transport to nascent vesicles, and vesicle neck division, and ESCRT is involved in the whole process (Thomas and Hurley, 2010). First is initiated by the ubiquitinated cargo aggregation on the endosomal membrane via HRS: STAM (ESCRT-0) and related adaptors (McCullough et al., 2013). After aggregation, HRS: STAM’s HRSPASP motif recruits TSG101 (Im et al., 2010) to form ESCRT-0 and ESCRT-I complexes, which localize the aggregated cargo to the bud neck and are responsible for carrying ubiquitinated proteins and PT/SAP motif-containing proteins to the lumen vesicles (Pegtel and Gould, 2019). During cargo transport, the C-terminal domain of VPS28 of ESCRT-Ⅰrecruits ESCRT-Ⅱ to form a complex responsible for membrane bud formation and cargo confine (Teo et al., 2006; Wollert and Hurley, 2010). Subsequently, ESCRT-Ⅲ is recruited by ESCRT-Ⅱ or ALIX to assist ESCRT-Ⅰ and ESCRT-Ⅱ complexes in catalyzing membrane division and factor recycling through VPS4 to finally form MVBs (Wollert and Hurley, 2010; McCullough et al., 2013).

ILVs and MVBs can also be generated in ESCRT-independent ways. This mechanism does not rely on ESCRT, but on lipids, ceramides, tetraspanins, or RAB proteins (Stuffers et al., 2009). Neutral sphingomyelinase (nSMase) can hydrolyze sphingomyelin to generate ceramide (Sindhu et al., 2021). Dreux et al. (Marlène et al., 2012) found that inhibition of nSMase can reduce the generation of ceramide and thus reduce the inward budding of the MVB membrane. This suggests that ceramides play an important role in the production of MVBs and ILVs. Ghossoub et al. (2014) also found that phospholipase D2 can hydrolyze phosphatidylcholine to produce phosphatidic acid, which can promote the inward budding of MVB membranes like ceramide, and encase specific proteins to generate ILVs. At present, the role of the tetraspanin protein superfamily in sorting Exos has also been elucidated. In human melanoma cells, CD63 can sort melanoma-associated proteins into human ILVs in the absence of ESCRT and ceramide (van Niel et al., 2011). In addition, it has also been reported that Rab31-FLOTs as an ESCRT-independent pathway play a very important role in the process of Exos generation (Denghui et al., 2021). RAB31 can be activated by multiple receptor tyrosine kinases (RTKs) through phosphorylation, and then drives the membrane budding of MVEs to form ILVs by binding to FLOTs proteins in the lipid raft microdomain. At the same time, RAB31 recruit TBC1D2B to the surface of MVEs to inactivate RAB7 to inhibit the fusion of MVEs and lysosomes, thereby facilitating the fusion of MVEs and cell membrane to release ILVs to form Exos.

#### 2.2.2 MVs

MVs originate directly from the plasma membrane, where they bud directly outward and detangle from the cell body to form vesicles. The important mechanism of MVs is the change in phospholipid distribution and cytoskeletal protein contraction (Cheng and Hill, 2022). Changes in calcium levels lead to the activation of calcium-dependent enzymes such as amino phospholipid translocation enzymes (Floppases and Flippases), resulting in the translocation of phospholipid serine (PS) outside the plasma membrane and internal structure imbalance of the plasma membrane, which then further induce budding of vesicles (Zwaal and Schroit, 1997; Leventis and Grinstein, 2010; van Niel et al., 2018). The contraction of cytoskeletal proteins mainly depends on the downstream signaling pathway mediated by the GTP-binding protein ADP-ribosylation factor 6 (ARF6). The combination of ARF6 and GTP promotes phospholipase D (PLD) activation, which in turn promotes the recruitment and activation of extracellular signal-regulated kinase (ERK), then ERK promotes myosin light chain kinase (MLCK) and myosin light chain (MLC) phosphorylation leading to actin and myosin contraction (Vandhana Muralidharan-ChariClancy et al., 2009). Some studies have reported that proteins involved in the biogenesis of Exos are also involved in the biogenesis of MVs (Rauch and Martin-Serrano, 2011; Joseph et al., 2012). Arrestin domain-containing protein 1 (ARRDC1) can mediate MVs formation, and protein tumor susceptibility 101 (TSG101) and apoptosis-linked gene 2 interacting protein (ALIX) of endosomal sorting complexes required for transport-I (ESCRT-I) which are involved in Exos biogenesis, are associated with ARRDC1 through their Pro-Ser-Ala-Pro(PSAP) and Pro-Pro-x (any amino acid)-Tyr (PPXY) motifs, then the complex is recruited to the plasma membrane leading to the release of MVs containing ARRDC1, TSG101 and so on (Rauch and Martin-Serrano, 2011; Joseph et al., 2012; Joseph et al., 2012; Abels and Breakefield, 2016; Anand et al., 2018).

#### 2.2.3 ApoBDs

ApoBDs are microparticles released during programmed cell death or late apoptosis. The formation process can be briefly summarized as three stages, initially chromatin agglutination followed by membrane blebbing and finally the decomposition of the cell contents into different membrane-encapsulated vesicles (Akers et al., 2013). In programmed death cells, nuclear DNA breaks into nucleosome fragments at nucleosome junctions and aggregates into condensed chromatin blocks under the nuclear envelope or in central heterochromatin regions. With the continuous accumulation of chromatin, the nuclear lamina breaks away, and the nuclear envelope breaks at the nuclear pores, forming nuclear debris. At the same time, in the process of programmed death, the cytoplasm is constantly concentrated, and the cell volume decreases due to continuous dehydration. Apoptotic cells undergo nuclear fragmentation to form chromatin blocks, which can then form apoptotic protrusions through budding, blebbing, *etc.* (Grant et al., 2019). The root of the protrusions can be constricted and shed to form vesicles of various sizes containing cytoplasm, organelles, and nuclear debris. The formed apoBDs are eventually removed by phagocytes (Cheng and Hill, 2022). Caspase-3 and ROCK1 are the key proteins that drive apoBDs (Leverrier and Ridley, 2001; Akers et al., 2013; Seo et al., 2022). Caspase-3 can cleave ROCK1 and then phosphorylate MLC, resulting in actin-myosin contraction (Charras et al., 2008a; Charras et al., 2008b). This promotes the detachment of the plasma membrane from the cytoskeleton. During the formation of apoBDs, membrane lipids are rearranged and phosphatidylserine (PS) is exposed to the surface of the membrane to promote macrophage recognition (Suzuki et al., 2013; Suzuki et al., 2022).

### 2.3 Uptake of EVs

Exosomes are secreted into the extracellular space by donor cells and alter the microenvironment through cell-to-cell interactions through fusion with the plasma membrane and subsequent endocytosis and release of cargo (Alptekin et al., 2022). EV uptake by cells appears to occur through a variety of endocytic pathways, including clathrin-dependent endocytosis and clathrin-independent pathways, such as fossa protein-mediated uptake, macropinocytosis, phagocytosis, and lipid raft-mediated internalization, and heterogeneous EV populations may enter cells by different route, depending on the proteins and glycoproteins found on the surface of the vesicle and target cells (Mulcahy et al., 2014). Specific protein-protein interactions can mediate EV attachment and uptake into cells. These proteins include tetratransmembrane proteins, integrins and immunoglobulins, proteoglycans, and lectins (Raposo et al., 1996; Théry et al., 1999; Tumne et al., 2009; Barrès et al., 2010; Christianson et al., 2013; Svensson et al., 2013). Endocytosis, including caveolin-dependent endocytosis, macropinocytosis, and lipid-raft mediated internalization, is a rapid and common pathway for EV uptake, which requires energy and cytoskeleton (Hao et al., 2007; Barrès et al., 2010; Feng et al., 2010).

In addition, it has been reported that viruses may have adopted existing EV-mediated communication pathways as their infection strategies, and exposure of phosphatidylserine groups on exosome surfaces has been observed in some studies, which may be one of the common targeting mechanisms of both exosomes and viruses (Feng et al., 2010; Fitzner et al., 2011; van Dongen et al., 2016).

### 2.4 EVs in clinical trials

At present, extracellular vesicles have been widely used and reported in clinical trials (Shah et al., 2018; Maacha et al., 2019; Elsharkasy et al., 2020; Hu et al., 2021; Russell et al., 2022).

The important role of extracellular vesicles as diagnostic biomarkers in the diagnosis of cancer (Krug et al., 2018; McKiernan et al., 2018), infection (Kornek et al., 2012; Delabranche et al., 2013), autoimmune disease (Burbano et al., 2019; Cuomo-Haymour et al., 2022) has been reported. Some of the protein, DNA, mRNA, miRNA, lncRNA, circRNA, and other cargo carried by extracellular vesicles in blood or tissue fluid will show specific increase or decrease under specific disease conditions, and can reflect disease degree and prognosis to a certain extent (Becker et al., 2016; Merchant et al., 2017; Wang et al., 2019; Thietart and Rautou, 2020).

Extracellular vesicles with therapeutic effects are widely used in the treatment of cancer (Shuen et al., 2022), cardiovascular disease (Lin et al., 2021; Reddel et al., 2021), inflammatory disease (Robbins and Morelli, 2014; Arab et al., 2020), degenerative disease (Liu et al., 2022a; Yin et al., 2022a). In addition, extracellular vesicles have been reported for the treatment of maternal and neonatal diseases (Mohammad et al., 2022Mohammad et al., 2022). We believe that clinical application of extracellular vesicles in more disease models is promising.

## 3 Native EVs for DODs

The efficacy of human embryonic mesenchymal stem cell exosomes in cartilage repair and MSC exosomes as an out-of-the-box, “cell-free” alternative to cell-based mesenchymal stem cell therapy was first reported in 2016 (Zhang et al., 2016a). In the same year, it was first reported that MSC exosomes promote myogenesis and angiogenesis *in vitro* and muscle regeneration *in vivo* models of muscle injury. Naturally secreted nano-carriers, exosomes, can be used as bioactive materials to improve the bioactivity of biomaterials. The use of hiPS-MSC-Exos in combination with β-TCP scaffolds to repair bone defects has also been reported for the first time, greatly advancing the progress in this field (Nakamura et al., 2015; Zhang et al., 2016b).

**FIGURE 2:**
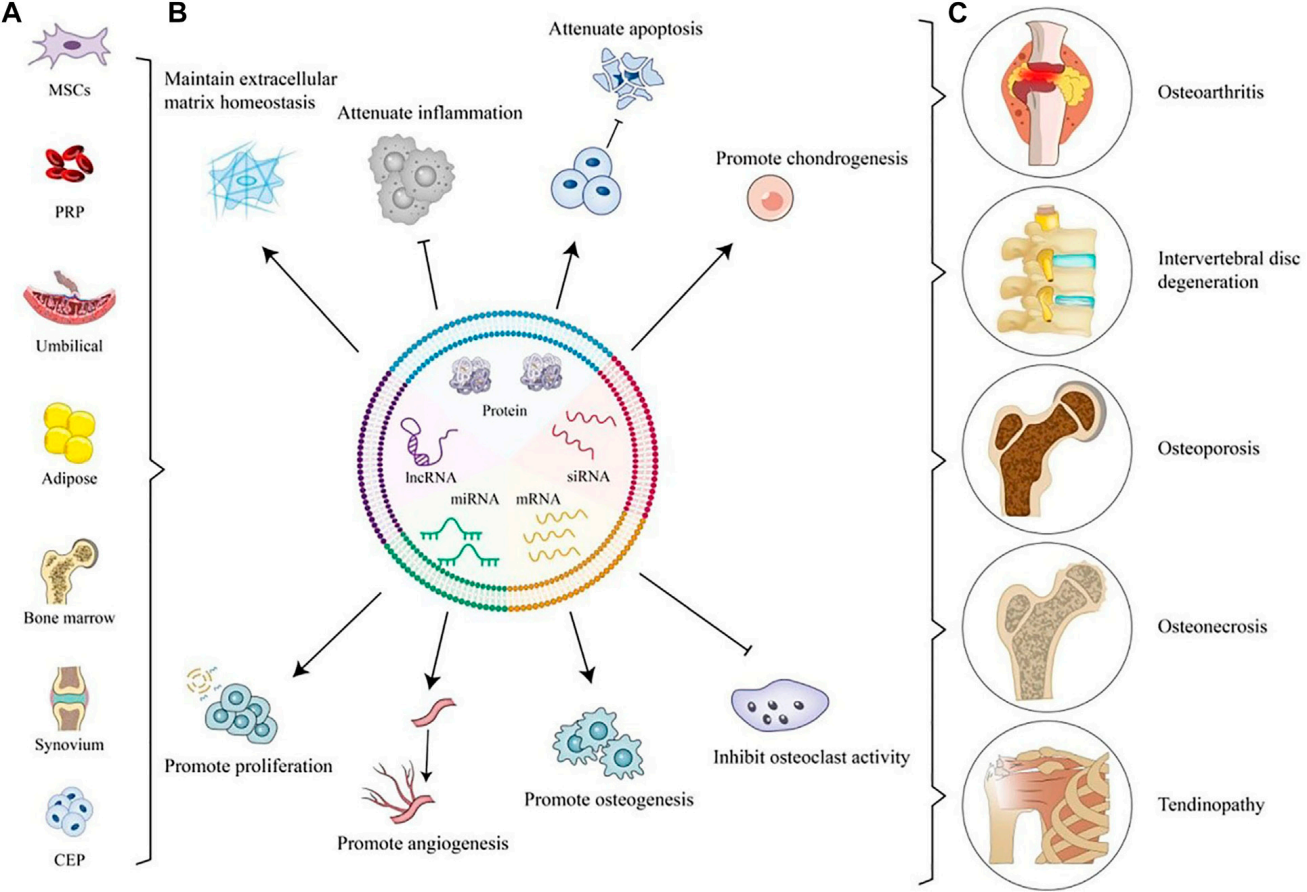
Application of Native EVs for OA, IVDD, OP, ON, and tendinopathy. **(A)** Sources of Native EVs. **(B)** Application of Native EVs to defect sites including maintaining ECM homeostasis, attenuating inflammation and apoptosis, promoting chondrogenesis, proliferation, angiogenesis, and osteogenesis, and inhibiting osteoclast activity. **(C)** Degenerative orthopedic diseases.

Currently, there are many articles on the use of native electric vehicles in DOD treatment, as shown in Table 1 and Figure 2. In terms of the source of EVs, people mostly use MSCs-derived EVs to treat DOD, but it has been reported that tissue-derived EVs also have satisfactory therapeutic effects (Lee et al., 2019; Lee et al., 2021; Yin et al., 2022b). In the future, the efficacy comparison of EVs from different sources can be further explored. In addition, EVs can encapsulate various proteins and RNAs to treat IDD, but little attention has been paid to the role of other cargo in EVs such as lipids and DNA in DOD treatment. Takahashi et al. (2017) proposed that EVs could play a role in maintaining the balance of the intracellular environment by eliminating damaged DNA and preventing abnormal innate activated immune responses. However, it is still unknown whether lipid or DNA in EV has a therapeutic effect on DOD. Moreover, pyroptosis and ferroptosis are new modes of death. Although it has been reported that those new modes of death are key factors in the pathogenesis of DOD (An et al., 2020; Tao et al., 2021; Zhao et al., 2021; Wang et al., 2022a; Yang et al., 2022), few papers have focused on how to use EVs to treat them, and the treatment of new forms of death can be further studied.

**TABLE 1:** Application of different sources of EVs in treating DODs.

Disease	EV function	EV source	Study conclusion	Study types performed	References
OA	Chondrogenesis	Human MSCs	Exos miR-92a-3p can promote cartilage development and maintain homeostasis by inhibiting WNT5A to treat OA	*In vitro* and *in vivo*	Feng et al. (2010)
	Maintenance of ECM homeostasis	Human PRP	PRP-EVs can promote the formation of ECM and chondrocytes and inhibit inflammation by activating the Wnt/β-catenin signaling pathway	*In vitro* and *in vivo*	Krug et al. (2018)
	Anti-inflammatory	Human MSCs	MSC-EVs can reduce cellular inflammation by inhibiting IL-1β-induced nitric oxide and MMP13	*In vitro* and *in vivo*	van Dongen et al. (2016)
OP	Maintain the stability of bone resorption and bone formation	Human UCMSCs	Osteogenesis-induced Exos derived from HucmSCs can participate in bone development and differentiation by highly expressing 221 microRNAs compared with Exos derived from unstimulated HucmSCs	*In vitro* and *in vivo*	Thietart and Rautou (2020)
		Human BMMSCs	LncRNA MALAT1 in EVs can enhance the activity of osteoblasts by MicroRNA-34c/satb2 axis	*In vitro* and *in vivo*	Chun-Yuan et al. (2019)
		Human USCs	USC-EVs can promote bone formation by transferring CTHRC1 and OPG proteins	*In vitro* and *in vivo*	Lin et al. (2021)
		Human BMMSCs	miR-27a delivered by MSCs-EVs could activate the Wnt/β-Catenin signaling pathway by inhibiting DKK2 and further inhibiting osteoclast activity and OP progression	*In vitro* and *in vivo*	Song et al. (2019)
		Human BMMSCs	MSCs can engulf ApoBDs through integrin αvβ3 and reactivate the ApoBDs-derived ubiquitin ligase RNF146 and Mir-3283p to inhibit Axin1, thereby activating Wnt/β-catenin pathway and playing an important role in the process of osteogenic differentiation	*In vitro* and *in vivo*	Reddel et al. (2021)
IDD	Anti-aging; Anti-apoptotic	Human CEPs	Compared with degenerated cartilage endplate cell-derived Exos, normal cartilage endplate cell-derived Exos have better efficacy in treating IDD by activating PI3K/AKT pathway	*In vitro* and *in vivo*	Nakamura et al. (2015)
		Human USCs	MATN3 enriched in USC-Exos induces NPCs proliferation, and ECM synthesis and alleviates IDD by increasing the phosphorylation of SMAD and AKT	*In vitro* and *in vivo*	Lee et al. (2019)
		Human Adipo	NAMPT delivered in sEVs could significantly improve physical activity and prolong the life span of aged mice by increasing NAD + levels	*In vitro* and *in vivo*	Yin et al. (2022a)
	Anti-oxidant; Anti-inflammatory	Human MSCs	MSC-Exos could alleviate ER stress-induced apoptosis by activating AKT and ERK signaling	*In vitro* and *in vivo*	Zhao et al. (2021)
		Mouse MSCs	Exos can suppress inflammatory mediators and NLRP3 inflammasome to suppress IDD; Exos may offer mitochondrial proteins to NP cells to alleviate IDD	*In vitro* and *in vivo*	Glyn-Jones et al. (2015b)
	Maintenance of ECM homeostasis	Mouse ADSCs	ADSCs-derived Exos can regulate matrix synthesis and degradation by regulating matrix metalloproteinases (MMPs) and inhibits pyroptosis by mitigating the inflammatory response	In vitroand *in vivo*	Ge et al. (2009)
		Human MSCs	MiR-17-5p delivered by H-sEVs can promote NPCs proliferation and ECM synthesis by targeting the TLR4/PI3K/AKT pathway	*In vitro*	Huang et al. (2017)
		Human BMMSCs	Cir0072464 in BMMSCs-evs can target miR-431 and promote the expression of NRF2 to inhibit NPC ferroptosis and facilitate matrix synthesis and proliferation	In vitroand *in vivo*	Ge et al. (2011)
ONFH	Angiogenesis and osteogenesis promotion	Human BMMSCs	BMMSCs-Exos promote osteogenesis by up-regulating the gene expressions of Bmp2, Bmp6, Bmpr1b, Mmp9 and Sox9	*In vitro*	Wang et al. (2020a)
		Human- iPS-MSC-	IPS-MSC-Exos can prevent ONFH by promoting angiogenesis and reducing bone loss via PI3K/AKT pathway	*In vitro* and *in vivo*	Ming-Li et al. (2021)
	Anti-apoptosis; Proliferation promotion	Human SMSCs	SMSC-Exos can enhance the proliferation and inhibit apoptosis of BMMSCs to treat ONFH	*In vitro* and *in vivo*	Yafei et al. (2017)
Tendinopathy	Maintenance of ECM homeostasis	Human TSCs	TSCs-Exos can treat tendinopathy by increasing the expression of TIMP3 and Col-1a1 and decreasing MMP-3 expression	*In vitro* and *in vivo*	Sambrook (1996)
		Human BMSCs	BMSCs-EVs can upregulate the expression of COL-1a1, SCX, and TNMD to treat tendinopathy	*In vitro*	Yue et al. (2018)
	Anti-inflammation	Human ASCs	ACS-EVs can inhibit early tendon inflammation by regulating the macrophage inflammatory response	*In vitro* and *in vivo*	Kimura et al. (2008)

OA, osteoarthritis; OP, osteoporosis; IDD, intervertebral disc degeneration; ONFH, osteonecrosis of the femoral head; MSCs, mesenchymal stem cells; PRP, platelet-rich plasma; UCMSCs, umbilical cord mesenchymal stem cells; BMMSCs, bone marrow-derived mesenchymal stem cells; iPS, induced pluripotent stem cells; SMSCs, synovial-derived mesenchymal stem cells; TSCs, tendon stem cells; ASCs, adipose-derived stem cells; ECM, extracellular matrix; EPCs, endplate chondrocytes; NPCs, nucleus pulposus cells.

### 3.1 OA

OA is a degenerative disease that worsens as people age and can lead to chronic pain and stiffness (Glyn-Jones et al., 2015a). The pathological characteristics of OA are very complicated, mainly involving cartilage degeneration, chondrocyte necrosis, matrix destruction, synovial hyperplasia, bone spurs formation, and subchondral osteomalacia (Xia et al., 2014; Abramoff and Caldera, 2020). At present, the research on the treatment of OA with EVs mostly focuses on their functions of chondrogenesis, anti-inflammation, and regulation of ECM as is shown in Table 1.

One of the important pathogenesis of OA is the imbalance between anabolism and catabolism of chondrocytes. Aging, trauma, biomechanics, obesity, and changes in biological rhythms can lead to chondrocyte hypertrophy or apoptosis, metabolic disorders, and cellular senescence, which in turn cause the destruction of cartilage homeostasis and induce OA (Glyn-Jones et al., 2015b). WNT5A, as a Wnt protein, plays an important role in the pathogenesis of OA, and it can mediate the destruction and degradation of cartilage. WNT5A has been reported to promote chondrocyte catabolism through noncanonical Wnt signaling, while also mediating cartilage destruction through the NF-kB pathway (Ge et al., 2009; Ge et al., 2011; Huang et al., 2017). EVs can enhance cartilage development and inhibit OA by targeting WNT5A. For example, Mao et al. found that miR-92a-3p in MSC-derived Exos could inhibit OA cartilage degradation by directly targeting WNT5A (Mao et al., 2018).

As an important part of articular cartilage, ECM can not only maintain the living environment of chondrocytes; but also play a supporting and nutritional role for chondrocytes. Its homeostasis plays a crucial role in maintaining the normal function of chondrocytes and timely responding to changes in the external environment. In the pathological setting of OA, loss of ECM leads to articular cartilage degeneration and articular structural deformity (Shi et al., 2019). EVs can promote the synthesis of cartilage matrix by up-regulating the expression of genes related to cartilage repair and syntheses such as SOX9, col2, and downregulate the genes of catabolic factors like MMP13, ADAMTS5, and MMP3 to inhibit degradation (Stella et al., 2017; Yafei et al., 2017; Hao et al., 2019; Shipin et al., 2019). Wnt/β-catenin and TGF-β signal pathways are common pathways involved in the synthesis of extracellular matrix and have been extensively studied (Wang et al., 2020a; Ming-Li et al., 2021). For example, PRP-derived EVs can promote the secretion of ECM by inhibiting the Wnt pathway and promoting the TGF-β pathway. Notably, this study also reported that PRP-EVs could promote chondrocyte proliferation and reduce the production of proinflammatory mediators (Liu et al., 2019).

In OA pathology, inflammatory or stress-induced signaling pathways may be activated in synovium and cartilage due to changes in the joint environment, leading to the production of cytokines, chemokines, adipokines, Toll-like receptor ligands, and other inflammatory mediators such as bone morphogenetic proteins (Elsa et al., 2022). These inflammatory mediators in turn lead to cartilage destruction and even ECM degradation through various signaling pathways such as ERK1/2 signaling (Robinson et al., 2016). Inhibiting the production of inflammatory mediators is one of the important mechanisms of native EVs in the treatment of OA. Zhang et al. found that MSC-Exos could activate AKT, ERK, and AMPK through adenosine receptors thereby inhibiting the production of NO and MMP13 (Shipin et al., 2019).

### 3.2 OP

OP is a systemic disease characterized by low bone mass and destruction of bone microstructure, resulting in increased bone fragility and susceptibility to fractures (Wang et al., 2009; Compston et al., 2022). It usually develops slowly over several years and will cause great physical and psychological pain to patients. OP is usually caused by the imbalance between bone resorption and bone remodeling. When bone resorption exceeds bone formation, it often leads to the loss of bone mass and eventually leads to OP (Sambrook, 1996; Kleerekoper and Sullivan, 2022). At present, studies have found that EVs could maintain the stability of bone resorption and bone formation, thus slowing down the development of OP.

MSCs-derived EVs have been discovered to inhibit OP by promoting osteoblast proliferation and differentiation (Yue et al., 2018; Do-Kyun et al., 2021). In a study, osteogenesis-induced Exos derived from HucmSCs can participate in bone development and differentiation by highly expressing 221 microRNAs compared with Exos derived from unstimulated HucmSCs, suggesting that the mechanism of EVs involved in osteogenic differentiation is dependent on microRNA delivery. Interestingly, this study also revealed that the expressions of hsa-mir-2110 and hsa-mir-328-3p were gradually increased during osteogenic differentiation and regulated the target genes related to osteogenic differentiation (Yahao and Xinjia, 2021). The specific mechanisms of these two miRNAs still need to be further elucidated. In addition to miRNAs, studies have reported that lncRNA MALAT1 in EVs can reduce osteoporosis by MicroRNA-34c/satb2 axis, suggesting that lncRNA can be used as a promising new regulator of osteogenesis in MSCs-EVs (Wang et al., 2020a). CTHRC1 and OPG proteins, as bone-promoting proteins, have also been found in USCs in recent years (Kimura et al., 2008; Chun-Yuan et al., 2019). Chen et al. found that USC-EVs can promote bone formation by transferring these two proteins and thus provide a novel approach to osteogenic therapy (Chun-Yuan et al., 2019). Osteoclasts are the only cells with the ability to dissolve bone tissue and play an important role in bone remodeling. Its abnormal formation and activity can lead to the development of osteoporosis. EVs can inhibit osteoclast activity by activating a variety of pathways such as OPG/RANKL/RANK and Wnt/β-Catenin signaling pathways. For example, miR-27a delivered by MSCs-EVs could activate the Wnt/β-Catenin signaling pathway by inhibiting DKK2 and further inhibiting osteoclast activity and OP progression (Yue et al., 2018). Exosomes secreted by vascular endothelial cells (EC-exos) exhibit more effective bone targeting than exosomes derived from osteoblasts or bone marrow mesenchymal stem cells (Song et al., 2019). Bone targeting EVs have been used to improve bone diseases including osteoarthritis, osteoporosis and bone tumors (Ren et al., 2022). It is worth noting that there are not many reports about other specific types of EVs other than Exos. MSCs can engulf ApoBDs through integrin αvβ3 and reactivate the ApoBDs-derived ubiquitin ligase RNF146 and Mir-3283p to inhibit Axin1, thereby activating Wnt/β-catenin pathway and playing an important role in the process of osteogenic differentiation (Dawei et al., 2018). The specific types of EVs such as apoBDs and MVs still need to be further studied.

### 3.3 IDD

IDD is a common bone degenerative disease, which is usually caused by the deterioration of one or more intervertebral discs in the spine with age, leading to back or neck pain. The intervertebral disc is a fibrocartilage structure, that is composed of three parts including the outer annulus fibrosus (AF), the inner nucleus pulposus (NP), as well as cartilaginous endplate (CEP) that connects the vertebral bodies (Zhichao et al., 2022). This complex structure plays an important role in reducing the stress caused by impact, absorbing shock for the spine, and protecting the nerves between the vertebrae. The pathogenetic changes of IDD are complex, including abnormal ECM synthesis and degradation, apoptosis, angiogenesis, inflammation, CEP calcification, and so on.

So far, a large number of literature have reported the therapeutic effects of native EVs in IDD (Krut et al., 2021). Senescence and apoptosis of NPCs, EPCs and DCs are considered to be one of the factors that promote the progression of IDD (DiStefano et al., 2022). Studies have shown that the cargo present in native-EV can target to reduce cell senescence and apoptosis, as well as promote the proliferation of NPCs, EPCs, and DCs (Jimenez et al., 1988; Yongjin et al., 2021). As is reported in the literature, native EVs may exert their therapeutic effect by acting on PI3K/AKT pathway, MAPK/ERK pathway, TGF-β pathway, and Wnt pathway (Jun et al., 2019; Tao et al., 2020; Shaoqian and Lei, 2021; Liwen et al., 2022a). For instance, cui et al. discovered that MiR-129-5p delivered by BMMSCs-derived EVs can decrease the apoptosis of NPCs and alleviate IDD by targeting the LRG1/p38 MAPK pathway (Shaoqian and Lei, 2021). Besides miRNA, proteins such as NAMPT and MATN3 encapsulated in native EVs can also alleviate IDD. MATN3 in USC-Exos is proved by Guo et al. that can activate TGF-β to promote the proliferation and suppress the senescence of NPCs (Guo et al., 2021). Adipo-sEVs can attenuate IVDD in rats by delivering NAMPT to restore senescent NPCs and EPCs (Yongjin et al., 2021).

Oxidative stress and inflammation are reported to play an important role in the development of IDD. The increased level of ROS and the production of inflammatory cytokines in IDD can activate multiple signaling pathways such as the NF-KB pathway, which can cause the damage of degenerative ID cells, and increased catabolism of extracellular matrix, leading to the deterioration of ID structure and function (Feng et al., 2017; Guang-Zhi et al., 2020; Hainian et al., 2020). The endoplasmic reticulum (ER) has been reported to be an organelle involved in oxidative stress and inflammation (Yan et al., 2021a; Dong et al., 2021). Inflammatory cytokines can activate ER stress by activating PERK and IRE1-α, thereby inducing apoptosis (Zhiwei et al., 2019; Wen et al., 2021). Exos can alleviate IDD by inhibiting ER stress. Liao et al. discovered that MSC-EVs could alleviate the advanced glycation end products (AGE)-induced ER stress of NPC cells and inhibit the excessive unfolded protein response (UPR) to alleviate apoptosis via the AKT/ERK pathway (Zhiwei et al., 2019). Another study found that USCs-exos could inhibit NPC cell apoptosis in a dose-dependent manner by inhibiting ER stress (HongFei et al., 2020). Mitochondria is another organelle closely related to oxidative stress and inflammation, and its damage will lead to the rise of intracellular ROS, promoting inflammation generation and cell death (Liang et al., 2022a). It has been reported that MSC-evs can attenuate mitochondrial dysfunction in NPC by supplementing mitochondria-associated proteins, thereby inhibiting ROS production and NLRP3 activation (Chen et al., 2019). These results suggest that both the ER and mitochondria are important targets for EV therapy.

The imbalance of homeostasis of ECM is reported to promote the progression of IDD. Hence, enhancing extracellular matrix synthesis and reducing its degeneration are essential for IDD. MMPs are a family of proteases involved in the degradation of ECM in various tissues throughout the body and play an important role in promoting IDD. Xing et al. found that ADSCs-EVs can reduce the activity of MMPs to maintain ECM homeostasis (Hongyuan et al., 2021). In addition, miR-17-5p delivered by MSCs-evs is reported to upregulate the synthesis of Col II and Aggrecan, as well as reduce the production of MMP13 and ADAMTS5 via TLR4/PI3K/AKT pathway (Zhi-Min et al., 2021). Cir0072464 in BMMSCs-evs can target miR-431 and promote the expression of NRF2 to facilitate ECM synthesis (Yu et al., 2022).

### 3.4 Other DOD

Osteonecrosis is the destruction of blood supply to the bone caused by different causes, which leads to the degeneration and necrosis of subchondral bone and finally results in the degenerative and destructive changes of the joint (Pavelka, 2000Pavelka, 2000). Since osteonecrosis mostly occurs in the femoral head, current research usually focuses on the treatment of osteonecrosis of the femoral head (ONFH) (Cohen-Rosenblum and Cui, 2019). It has been reported that the angiogenic and apoptotic activities of bone microvascular endothelial cells (BMESs) are altered in patients with osteonecrosis of the femoral head, which suggests that ONFH could be treated by promoting angiogenesis and increasing osteogenesis (Weinstein, 2011; Noam et al., 2019). BMMSCs have multi-directional bone tissue differentiation potential, so they are widely used in the field of ONFH treatment. However, researchers have found that although BMMSCs have good characteristics of easy to extract and obtain immunogenicity, their proliferation, and osteogenic potential gradually decline with age. Guo et al. (2016) discovered the integration of BMSCs and SMSC-EVs would play a better role in proliferation and anti-apoptosis, which suggested that MSCs combined with EVs therapy had better application potential. EVs therapy alone has similarly been reported to have the potential to treat ONFH. BMMSCs-Exos can promote osteogenesis by up-regulating the gene expressions of Bmp2, Bmp6, Bmpr1b, Mmp9, and Sox9 and activate osteoblast differentiation, and in the transforming growth factor-β/bone morphogenetic protein pathways to inhibit ONFH (Shanhong et al., 2022). The PI3K/AKT pathway is another pathway in which EVs have been reported to be potentially involved in the treatment of ONFH (Jinhui et al., 2022; Xiaolin et al., 2023). Liu et al. revealed that iPS-MSC-Exo could promote the proliferation, migration, and tube-forming of endothelial cells via PI3K/AKT pathway (Xiaolin et al., 2023).

Tendinopathy, characterized by degenerative changes in cellular structure along with tendon mechanical properties, is a painful and swelling disease that can finally lead to the rupture of the tendon (Woodley et al., 2006; Millar et al., 2021). ECM disruption is one of the important pathogenesis of tendinopathy. MMP, as one of the proteolytic enzymes that degrade ECM, can be activated by inflammatory factors or mechanical stress to hydrolyze ECM and promote tissue damage (Godoy-Santos et al., 2012; Baroneza et al., 2014). However, TIMP, a natural inhibitor of MMP, can inhibit tissue degradation and antagonize MMP (Thornton et al., 2008). Wang et al. found that TCS-EV could reduce the expression of MMP-3 and increase the expression of TIMP3 and Col1a1, thereby improving the biomechanical properties of the tendon (Yunjiao et al., 2022). In another study, BMMSC-EVs can promote tendon repair by upregulating tenogenic differentiation and tendon matrix formation-related genes such as Col-1a1, SCX, and so on (Zhengzhou et al., 2019). Inflammation also contributes to the progression of tendinopathy and EVs can exert anti-inflammatory effects through a variety of mechanisms (D’Addona et al., 2017; Dakin et al., 2017). For example, inflammation-stimulated ASCs can reduce the inflammation response of the tendon by inhibiting NF-KB activity and the expression of proinflammatory cytokine II 1b (Shen et al., 2020).

## 4 EV engineering strategies

In contrast to transplantation of exogenous Mesenchymal stem cell (i.e., cell therapy), EVs offer a good alternative to a regimen, that is non-proliferative, immunogenic, and easier to store and transport than cells, it overcomes the problems of uncontrollability, imprecise induced differentiation and low survival rate of stem cells (Liu et al., 2022a; Sun et al., 2022a; Erwin et al., 2022; Zeng et al., 2022). However, native EVs still have some problems in practical application, such as insufficient tissue targeting, easy to be cleared, not lasting effect, limited curative effect, considerable difficulty to monitor the distribution *in vivo*, and limited amount of phagocytosis by target cells, *etc.* (Xu et al., 2022a; Hiroaki et al., 2022; Zeng et al., 2022; Yujie et al., 2023). Therefore, engineered EVs, as a therapeutic strategy to overcome the above-mentioned defects, has been widely developed and applied in recent years. For example, researchers can modify EV surface receptors to achieve better tissue targeting of EVs, and provide a sustained-release strategy for engineered EVs by materials science methods that can extend the duration of EV action; EV efficacy can be enhanced by modifying EV cargo, loading small molecule drugs or bioactive proteins and factors, and better monitoring of EV distribution can be achieved by engineering modifications Engineering modification increases the number of EV engulfed by target cells (Andaloussi et al., 2013; Zhang et al., 2021b; Bie et al., 2022; Zeng et al., 2022; Liubov and Isaac, 2023).

**FIGURE 3:**
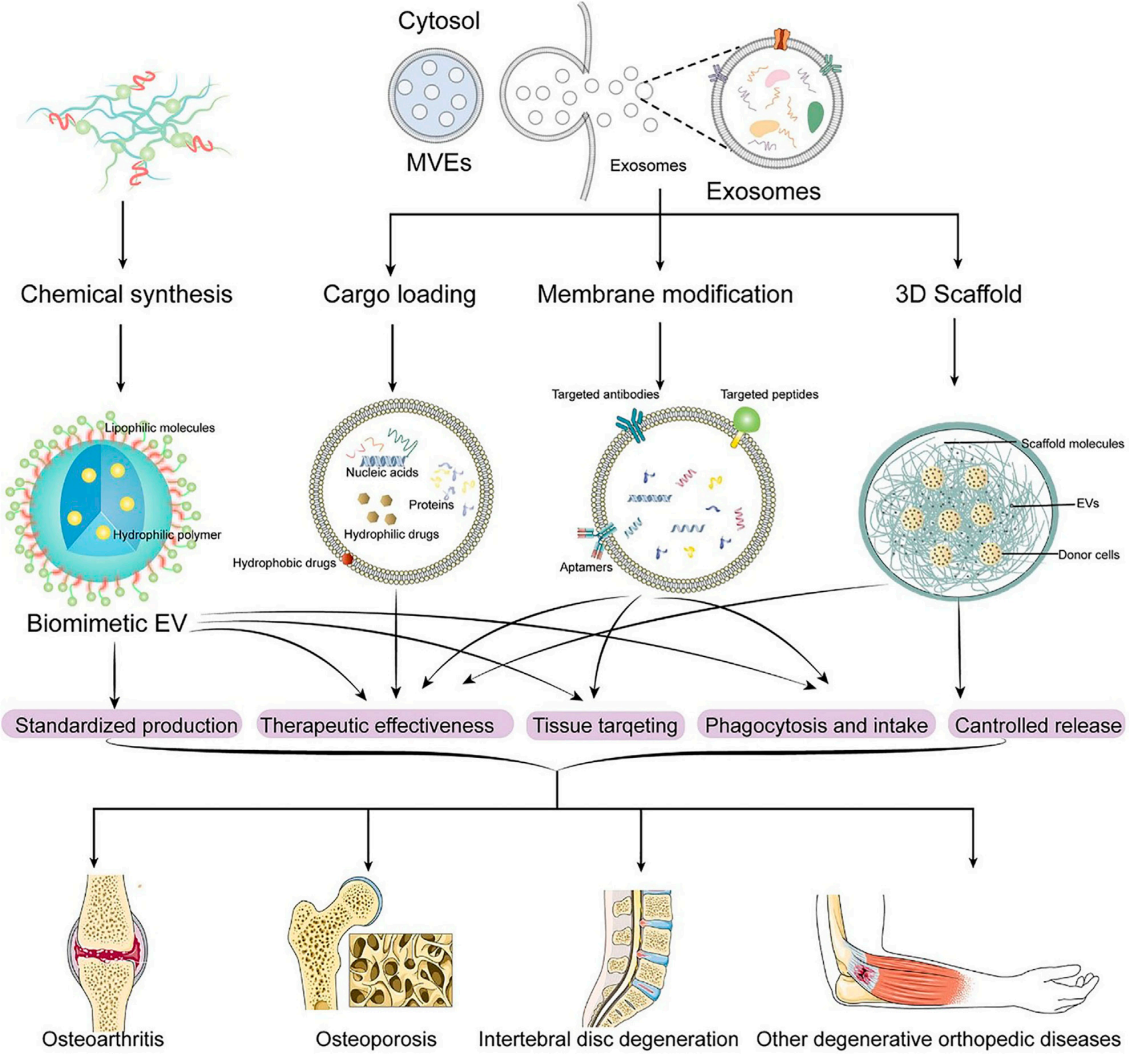
Engineering methods of EVs and application of engineered EVs in DODs.

We will introduce the engineered EVs from different EV engineering methods and the application of engineered EVs in different degenerative orthopedic diseases, as is shown in Table 2 and Figure 3.

**TABLE 2:** Application of engineered EVs for DODs.

Disease	EV function	EV engineering strategy	Study conclusion	Study types performed	References
OA	Chondrogenesis	Electroporation	Engineered EVs targeted KGN delivery to SF-MSCs, increased its effective concentration in cells, and strongly promoted chondrogenesis in SF-MSCs *in vitro* and *in vivo*	*In vitro* and *in vivo*	Weinstein (2011)
		Gene transfection	Engineered EVs with high expression of miR-140-5p enhanced ACs proliferation and migration *in vitro* without compromising ECM secretion	*In vitro* and *in vivo*	Liang et al. (2022a)
	Regulating ECM	Electroporation	Engineered EVs efficiently encapsulated miR-140, resulting in reduced protein levels of MMP-13 and Adamts-5 in cartilage tissues	*In vitro* and *in vivo*	Liubov and Isaac (2023)
OP	Osteogenesis	Co-incubation; Electroporation	Engineered EVs modified with bone-targeting peptides silenced the Shn3 gene in target cells, thereby increasing SLIT3 production and thereby promoting blood vessel formation, especially H-type blood vessel formation	*In vitro* and *in vivo*	Zhiwei et al. (2019)
		—	BMSCs-Exos loaded with miR-29a can transport miRNA-29a to HUVECs and increase trabecular bone mass in mice	*In vivo*	O’Loughlin et al. (2017)
		Co-Incubation	Intravenous administration of STExo-Aptamer complex can target bone and promote osteogenesis through miR-26a, enhance bone mass in OVX mice and accelerate bone healing in a mouse model of femur fracture	*In vitro* and *in vivo*	Shenglong et al. (2021)
		Co-Incubation	EVs target bone through alendronate/hydroxyapatite binding and promote the growth and differentiation of murine MSCs	*In vitro* and *in vivo*	Yongzhi et al. (2022)
	Vascularization	Co-incubation; Electroporation	EVs modified with bone-targeting peptides silenced the Shn3 gene of osteoblasts to enhance osteogenic differentiation, and decreased RANKL expression to inhibit osteoclast formation	*In vitro* and *in vivo*	Zhiwei et al. (2019)
IDD	Anti-apoptosis; Anti-aging	Gene transfection	After downregulation of miR-31-5p in MSC-EV, the protective ability to reduce apoptosis and calcification of EPCs through regulation of miR-31-5p/ATF6/ER stress pathway was decreased	*In vitro* and *in vivo*	Chen et al. (2022)
		Gene transfection	Lenti-Sphk2-Exos was engineered to transport Sphk2 to activate autophagy and reduce the aging in NPC	*In vitro* and *in vivo*	Yu et al. (2021)
	Anti-oxidant; Anti-inflammatory	Gene transfection	Cavin-2-engineered EVs can restore the cellular uptake of NPC and show better therapeutic effects on injured NPC	*In vitro* and *in vivo*	Shi-Cong et al. (2017)
		Electroporation	Engineered EVs loaded with FOXF1 reduced the expression of inflammatory cytokines (interleukin IL-1β, IL-6) in NPC	*In vitro* and *in vivo*	Herrmann et al. (2021)
	Regulating ECM	Electroporation	Engineered EVs loaded with FOXF1 reduced the expression of matrix metalloproteinase 13 (MMP13) in NPC	*In vitro* and *in vivo*	Herrmann et al. (2021)
	Other function	Gene transfection	miR-15a transfected NP-EVs affected chondrogenesis, differentiation, and protein balance through PI3K/Akt and Wnt3a/β-catenin axes	*In vitro*	Waters et al. (2019)
		Extrusion	Engineered EVs generated at 0.5 MPa inhibited angiogenesis by transferring overexpressed (miR)-140-5p into endothelial cells and regulating the downstream Wnt/β-catenin pathway	*In vitro* and *in vivo*	Liwen et al. (2022b)
Other DOD	—	—	—	—	—

OA, osteoarthritis; OP, osteoporosis; IDD, intervertebral disc degeneration; MSCs, mesenchymal stem cells; SF-MSCs, synovial fluid-derived mesenchymal stem cells; BMMSCs, bone marrow-derived mesenchymal stem cells; ECM, extracellular matrix; EPCs, endplate chondrocytes; NPCs, nucleus pulposus cells.

### 4.1 Loading cargos

EV is a delivery tool with good biocompatibility, which can load bioactive molecules to achieve efficient intracellular delivery. Loading drugs, proteins, RNA and other molecules into EV can enable EV to obtain specific therapeutic functions. The engineered EVs usually have better performance and therapeutic potential than native EV. The loading methods of these EV cargoes can generally be divided into passive loading and active loading (Luan et al., 2017; Zhang et al., 2021b; Zhang et al., 2021b).

#### 4.1.1 Active loading

##### 4.1.1.1 Co-incubation

Co-incubation is a method for small-molecule chemical compounds being introduced into EV (Zhang et al., 2021b). The co-incubation of different molecules with EVs under appropriate physical and chemical conditions and time will enable EVs to be endowed with corresponding functions. MSCs, upon initiation with paclitaxel (PTX), can generate MVs with strong antitumor activity through their ability to absorb and then release the drug (Luisa et al., 2014). A study reported that incubation of 15 cc-siRNA molecules per EV for 1 h at 37°C in 100 μL promoted concentration-dependent silencing of human antigen R (HuR) (a cancer therapeutic target) in EV-treated cells (O'Loughlin et al., 2017). EVs was mixed with equal-volume chito-oligosaccharide (COS), incubated at 37°C for 1 h, centrifuged at 120,000 × *g* for 60 min, and then suspended with 200 μL PBS to obtain EVS-COS conjugate which can promote cartilage repair and relieve osteoarthritis (Shenglong et al., 2021). To obtain EVs that could target osteoblasts, one study reported adding 10 μL EV and 90 μL bone-targeting peptide to 100 μL PBS and incubating the mixture overnight at 4 °C. Unbound bone-targeting peptides were then removed by hypercentrifugation at 10 5 × *g* for 70 min at 4°C and washed and resuspended with PBS (Yongzhi et al., 2022).

##### 4.1.1.2 Gene transfection

Gene transfection is a common strategy to modify EV characteristics at genetic level (Zhang et al., 2021b). It can be achieved by donor cell lentivirus transfection, adenovirus transfection, electroporation, microfluidic and other methods. For example, genetic engineering and co-transfection of parental cells are combined to create EVs that exhibit RVG peptide on their surface, target α7-nAChR, and enrich neutral lysozyme variants with higher specificity and beta-amyloid peptide (Aβ) degradation efficacy for the treatment of Alzheimer’s disease (AD) (Yu et al., 2021). Transfecting human adipose stem cells (hASCs) with microRNA-146a to improve and control EV composition is a viable therapeutic wound repair therapy (Waters et al., 2019).

Gene transfection is a common engineering strategy in the study of degenerative orthopedic diseases. Lentivirus transfection is the most commonly used method. A study on OA showed that EV transfected by lentivirus from human synovial mesenchymal stem cells overexpressing miR-140-5p enhanced chondrocyte proliferation and migration without affecting ECM secretion (Shi-Cong et al., 2017). In another study, lenti-SpHK2-engineered EVs (Lenti-Sphk2-Exos) obtained by lentivirus transfection were compared with Lenti-shSphk2 engineered EVs. Lenti-Sphk2-Exos that overexpress Sphk2 were found to improve IVDD by activating the phosphatidylinositol 3-kinase (PI3K)/p-AKT pathway and intracellular autophagy in nucleus pulposus cells (Liwen et al., 2022b).

#### 4.1.2 Passive loading

##### 4.1.2.1 Passive diffusion

Passive diffusion is widely used in the loading of lipid-soluble small molecules. By directly incubating EVs or mother cells with drugs, drugs can be diffused into EVs along concentration gradients (Andaloussi et al., 2013; Herrmann et al., 2021). This approach has been used sparingly in studies of engineered EVs for the treatment of degenerative orthopedic diseases.

##### 4.1.2.2 Electroporation

Electroporation (EP) is a commonly used strategy to increase cellular permeability for intracellular cargo delivery or to use electric fields for cell membrane disruption (Choi et al., 2022). Electroporation of EVS is an economical and effective method, so it is widely used in the research of degenerative orthopedic diseases. A previous study reported that macrophage-derived EVs and potent anti-inflammatory immune modulator interleukin-10 were integrated by electroporation for the treatment of rheumatoid arthritis (RA) (Tang et al., 2022a). EVs modified with the milk-derived EV-binding peptide iRGD (targeted to lung adenocarcinoma cells) were loaded with paclitaxel (PAC) and used for tumor therapy by electroporation (Chen et al., 2022). Moreover, EP also promotes feasibility and efficiency of CRISPER-Cas9 editing. Cas9 RNP was loaded into purified EVs isolated from hepatic stellate cells by electroporation for tissue-specific gene therapy in liver diseases (Tao et al., 2023). EVs with a total protein concentration of 12 μg and 100 nM KGN were electroporated using a Neon electroporation system with a pulse width set to 10 ms, and EV samples were then diluted in PBS and centrifuged to remove unloaded KGN (Xiao et al., 2020). Engineered FOXF1-loaded EVs were obtained by batch electroporation using Neon transfection system MPK5000 at 1,425 V, 30 ms and one pulse, which can be used as a non-viral reprogramming tool for human nucleus pulposus cells (Tang et al., 2022b).

##### 4.1.2.3 Mechanical methods

Mechanical methods include, but are not limited to, extrusion, freeze-thaw and ultrasonic treatment. For extrusion, a previous study reported that the EV-loading of hydrophilic porphyrins was significantly increased by using syringe-based small hand-held extruders (Avanti Lipids) at 42°C, where each sample was extruded 31 times (Fuhrmann et al., 2015). Repeated freeze-thawing mediated catalase loading into vesicles provides a strategy of significant neuroprotection in an *in vitro* and *in vivo* model of Parkinson’s disease (PD) (Haney et al., 2015). Mild ultrasound treatment was used to encapsulate curcumin and albumin into EVs, inhibit and reverse LPS-triggered expression of the inflammatory transcription factor NF-κB, and effectively block and reverse skin inflammation *in vivo* in mouse and rat models (Yerneni et al., 2022).

### 4.2 Modification of EVs

The modification of EV includes the modification of EV membrane, such as the receptor loading and targeted modification of EV membrane, and the artificial bionic nano vesicle system designed based on the material science method.

#### 4.2.1 EV membrane modification

The transformation of EV membrane is commonly achieved by chemical methods and genetic engineering. Previous studies have shown that lentivirus transfection of mesenchymal stem cells (MSCS) with high PD-L1 expression derived MSC-sEVs-PD-L1 EV has the ability to regulate various activated immune cells to an immunosuppressive state, and can be used for autoimmune diseases (Xu et al., 2022b). For chemical modifications, a study reported that EVs with glycosyl phosphatidylinositol-anchored antibiotin protein (GPI-Av) on the surface of donor cells were genetically engineered to demonstrate the specific binding and uptake of biotinylated ligand-bound carbonic anhydrase IX (CAIX) expressing cells for tissue-specific delivery (Sabani et al., 2022). Moreover, the EV membrane was modified with hexadecimal oligarginine (a cell-penetrating peptide) to induce actin-dependent endocytosis and macropinocytosis pathways, which resulted in efficient cellular uptake and significant cancer cell killing boron neutron capture therapy (BNCT) activity (Hirase et al., 2022).

#### 4.2.2 Biomimetic EV

Biomimetic EV nanoparticles, including nanovesicles coated with cell-membrane and nanoparticles synthesized by organic matter, are an emerging and promising solution developed based on materials science, which can overcome the obstacles such as low separation rate, low drug payload, and potential safety problems in the process of EVs being used as therapeutic drugs and delivery vesicles (Lu and Huang, 2020). For cell-membrane coated biomimetic EV, a research reported the use of biomimetic outer membrane vesicles (OMVs) as vaccine vectors against infection and cancer (Mat Rani et al., 2022). In another research, biomimetic vesicles are designed by camouflaging catalytic DNA machinery of breast cancer cell membrane, which enables molecular classification of circulating EVs to diagnose breast cancer subtypes through isotype recognition (Cao et al., 2022). In addition, membrane-derived bacterial extracellular vesicles (BEVs) have been reported to have great potential as novel drug delivery platforms (Han et al., 2021; Liu et al., 2022b). For biomimetic EV synthesized by organic matter, seven key mirnas used for protection were loaded into hyaluronic acid-polyethylenimine nanoparticles in a specific proportion to achieve *in vitro* mimicry of EVs, which can alleviate sepsis in mice and cynomolgus monkeys (Yinan et al., 2022). sPLA2 I-loaded micellar NPs (sPLA2), prepared by incorporation of lipid-based sPLA2i, thioideamide-PC into nanoscale phospholipid micelles, have been demonstrated to be effective in suppressing inflammatory signaling and attenuating OA progression after direct delivery to the knee joint (Wei et al., 2021b). In another study, DSPE-PEG-NHS and tetracycline were mixed in CHCl3, triethylamine was adjusted for PH, stirred at room temperature, dried for film with lecithin and cholesterol, NaHCO3 shock, phacoemulsification, stabilized dialysis, Finally, tetracycline modified NaHCO3 nano-liposomes (NaHCO3-TNL) with the ability to target the bone surface and prevent osteoporosis in response to the external secretory acidification of osteoclasts were obtained (Lin et al., 2020a).

#### 4.2.3 3D scaffold-based controlled release

At present, with the continuous development of materials science, biomaterial scaffolds have been widely used in tissue engineering and regenerative medicine. The materials of 3D scaffolds include natural compounds and synthetic polymers. 3D scaffolds mainly include hydrogels, particles and porous solids, and cross-linking and bioprinting are the main manufacturing methods (Chenyang et al., 2021).

EV therapy based on 3D scaffolds has become a research hotspot of EV delivery pathway. However, there is currently no precise definition of engineered EVs in academia, so whether hydrogel drug delivery is an engineering means for EVs or merely an improvement on the native EV drug delivery strategy remains controversial. Due to the complex and diverse materials science approaches currently applied in hydrogel-loaded EVs papers, many of them have reported improved EV performance and increased therapeutic potential (Cheng et al., 2021; Born et al., 2022; Sun et al., 2022b; Wang et al., 2022b; Xu et al., 2022c; Kenny et al., 2022; Man et al., 2022; Yunhui et al., 2022). So, we will consider 3D scaffold—based controlled release as a special type of engineering strategy for EV is specifically described here. But the rest of the narrative will focus on engineered EVs obtained through other methods.

In the relevant research of degenerative orthopedic diseases, hydrogel 3D scaffolds are mainly used. Hydrogels are cross-linked three-dimensional polymer networks that play an important role in tissue regeneration applications (Grundke et al., 2015; Eelkema and Pich, 2020; Stengelin et al., 2022). In recent years, there have been many studies on EVs loaded by hydrogels. Hydrogels with excellent properties can help EVs achieve slow release and targeted delivery and promote EV production when loading donor cells (Grundke et al., 2015; Eelkema and Pich, 2020; Ju et al., 2022; Stengelin et al., 2022; Looij et al., 2023). However, due to the characteristics of the disease model, the hydrogel-loaded EV release system has been studied more in the fields of skin injury, spinal cord injury and degenerative orthopedic diseases, but less in the fields of tumor and circulatory system diseases (Silva et al., 2018; Luo et al., 2021; Zhiwei et al., 2021; Fan et al., 2022; You et al., 2022).

## 5 Engineered EVs for degenerative OD treatment

In recent years, the number of studies on the treatment of degenerative orthopedic diseases with engineered EVs has increased explosively. Thanks to the improvement of disease mechanism related research and the rapid development of pharmacology and materials science.

### 5.1 OA

In OA patients, the abnormal expression of microRNA (miRNA) is associated with cartilage lesions (Glyn-Jones et al., 2015b). Engineered EVs designed for elevated or silenced miRNA expression have been shown to effectively inhibit inflammation, matrix degradation, apoptosis, chondrocyte proliferation and migration, and improve chondrocyte matrix secretion. In the treatment of degenerative osteoarthropathy, it shows better performance and broader prospects than native EV or drug direct treatment (Liang et al., 2020; Esmaeili et al., 2021; Sun et al., 2022b; Wan et al., 2022).

Engineered EVs can provide combinatorial effects between their natural cargo and externally added drugs or biomaterials, while also directing the treatment toward the desired goal. Loading bioactive molecules and drugs into electric vesicles for the treatment of OA is a common method for electric vesicle engineering in the field of OA (Zhang et al., 2022; Zhuang et al., 2022).

For promoting chondrogenesis and cartilage regeneration, Kartogenin (KGN), as yiz red, has been found to induce the differentiation of SF-MSCs into chondrocytes *in vitro* and *in vivo*. After being loaded with engineered EVs loading of MSC-binding peptide E7, it can target SF-MSCs and increase their effective concentration in cells. It can strongly promote chondrogenesis of SF-MSCs both *in vitro* and *in vivo*, showing a more significant effect than KGN direct treatment (Xiao et al., 2020).

To regulate the microenvironment of cartilage tissue, the engineered EVs obtained by gene transfection can effectively wrap miR-140, and the binding of chondrocyte affinity peptide (CAP) to EV membrane gives it the ability to specifically target chondrocytes. Then, by reversing IL-1β-induced activation of MMP-13, which resulted in the reduction of MMP-13 and Adamts-5 protein levels in cartilage tissue, OA progression was significantly inhibited, showing a more significantly enhanced effect than simple use of native EV (256). In order to overcome the side effect of MSC-EV reducing ECM secretion, miR-140-5p was highly expressed in EVs by gene transfection, thus enhancing the proliferation and migration of ACs without destroying ECM secretion (Shi-Cong et al., 2017).

To solve the problem of limited distribution and insufficient bioavailability of MSC-EV after an intraarticular injection, MSC-sEV was modified with a new cationic amphiphilic polymer ε-polylysine-polyethylene-stearyl phosphatidylethanolamine (PPD) to reverse the surface charge of MSC-sEV. Results showed that PPD-sEV regulated chondrocyte absorption and homeostasis more effectively than unmodified MSC-sEV, significantly enhancing chondrogenic absorption, cartilage penetration and joint retention (Feng et al., 2021). To address the issues of rapid joint clearance of drugs (i.e., short half-life) and therapeutic targets deep in the cartilage that drugs cannot reach, one study used micellar nanoparticles loaded with phospholipase a2 inhibitors to penetrate deep into the cartilage matrix, reduce inflammation, prolong joint space retention, *In vitro* model of cartilage explants and two animal models of OA showed a significant effect of slowing down the progression of OA (Wei et al., 2021b).

In addition to drug delivery vesicles, perhaps engineered electric vesicles could also be used as non-viral gene-editing tools for osteoarthritis treatment. However, special attention should be paid to its immunogenicity, biocompatibility and potential tumorigenicity (Shi-Cong et al., 2017).

### 5.2 OP

The pathogenesis of osteoporosis includes excessive bone resorption, insufficient bone formation and insufficient vascularization (Liang et al., 2022b). Engineered EVs carrying therapeutic molecules are promising as alternative therapies for osteoporosis, requiring the design of specific functionalized vesicles and appropriate engineering strategies.

In order to enhance osteogenesis, studies have reported that EVs modified with bone-targeting peptide can specifically deliver siRNA to osteoblasts, silence Shn3 gene, and reduce the expression of RANKL in osteoblasts, thus showing more significant functions of enhancing osteogenic differentiation, inhibiting osteoclast differentiation, and promoting blood vessel formation than native EV (Yongzhi et al., 2022). In another study, BMSCS-Mir-29a-ExOS-treated mice showed significant increases in bone mineral density (BMD), trabecular volume (BV/TV), and trabecular number (Tb. N) compared with BMSCs-Exos and PBS-treated mice. These results indicate that engineered EVs have a strong ability to enhance bone mass in mice (Lu et al., 2020).

*In vivo* treatment of osteoporosis, intravenous injection is often used. Therefore, how to deliver EVs to bone tissues in a high concentration, tissue-specific and tissue-targeted manner, rather than aggregation with other organs or tissues, is a key issue for EV engineering in osteoporosis treatment. A study reported that although bone marrow stromal cell (ST) -derived EVs (STExos) significantly enhanced osteoblast differentiation of BMSCs *in vitro*, STExos accumulated in the liver and lung after intravenous administration, resulting in ineffective improvement of osteoporotic phenotype in a mouse model of postmenopausal osteoporosis induced by ovariectomy (OVX) (Zhong-Wei et al., 2019). The StexO-aptamer complex was combined with BMSC-specific aptamer on the surface of STExo to test its ability to enhance bone mass in OVX mice and accelerate bone healing in femur fracture mouse model (Zhong-Wei et al., 2019). Another study reported the use of Ale-N3 to modify EVs derived from murine mesenchymal stem cells to generate Ale-EVs system with high affinity for bone by utilizing the specific binding properties of alendronate and hydroxyapatite, achieving the purpose of improving tissue-specific targeting of EVS (Wang et al., 2020b).

Synthetic nanovesicles have also been used in the treatment of OP. To inhibit osteoclast function and promote osteoclast apoptosis, NaHCO3-coated tetracycline modified nanoliposomes (NaHCO3-tnl) can spontaneously generate “nano-sacrifice layer” on the bone surface. Once H+ is secreted by osteoclasts, NaHCO3-TNL releases the bicarbonate radical (HCO3-), which neutralizes the acidic environment (Lin et al., 2020a).

### 5.3 IDD

In recent years, some studies have shown that engineered electric vehicles play a more important and superior role than native electric vehicles in protecting NPC and delaying the progress of IDD. Engineered EVs for IDD therapy are mainly obtained through genetic engineering (Zhang et al., 2021c; Zhiwei et al., 2021; Luo et al., 2022). The second most common method is mechanical method (Sun et al., 2020; Tang et al., 2021).

Targeting EV endocytic pathways and NPC pyroptosis, a previous study has reported that, compared with the EVs of the negative control group, the modified engineered EVs obtained by Cavin-2 gene editing in MSCs significantly increased the uptake rate of EVs by TNF-α-treated NPCs, reduced the death of NPCs, and delayed the progress of IDD *in vitro* organ models (Zhiwei et al., 2021). For anti-oxidant and anti-inflammatory, transfection of NPC with FOXF1 by engineered EVs can significantly change gene expression by up-regulating FOXF1 and KRT19 and down-regulating IL-1β, IL-6, MMP13, and NGF, which offers a potential approach for the treatment of IVD degeneration and associated back pain (Tang et al., 2021). Aiming at regulating cartilage differentiation, engineered EVs overexpressing miR-15a by gene transfection can promote chondrogenic differentiation of nucleus pulposus mesenchymal stem cells by PI3K/Akt and Wnt3a/β-catenin axes to down-regulating MMP-3 (Zhang et al., 2021c). Targeting the autophagy and senescence pathways in nasopharyngeal carcinoma, overexpression of EVs Sphk2 secreted by transgenic chondroendplate stem cells (CESCs) can penetrate the ring of fibers (AF), transport Sphk2 to nucleus pulposus (NPC), activate PI3K/AKT signaling pathway, regulate autophagy/senescence in nasopharyngeal carcinoma *in vivo* and *in vitro*, and prevent intervertebral disc degeneration. The performance is better than that of native CESC-EVs (Luo et al., 2022). Moreover, EVs can also be designed for anti-angiogenesis. EVs produced by notochord cells under extrusion load overexpress microRNA (miR)-140-5p, which can transfer to endothelial cells and regulate downstream Wnt/β-catenin pathway to inhibit angiogenesis (Sun et al., 2020).

Engineered EVs approach can also be used to explore the mechanisms related to IDD and its treatment. For example, by silencing or overexpressing miR-129-5p and SOX4 in human mesenchymal stem cells EVs (hBMSC-EVs), it was demonstrated that hBMSC-EVs enhanced the proliferation of NPC and the synthesis of ECM by delivering miR-129-5p into NPCS, targeting SOX4 and inactivating the Wnt/β-catenin axis (Wang et al., 2021). Moreover, By changing the expression of miR-194-5p in MSC-EVs, the mechanism is elucidated by which MSC-EVs can effectively enhance proliferation and osteogenic differentiation, and reduce the apoptosis of TNF-α-treated NP cells (Zhongyi et al., 2021). When microRNA-31-5p levels in mesenchymal stem cell-derived EVs (MSC-EVs) were downregulated, the therapeutical effect of MSC-EVs in IVDD was inhibited (Lin et al., 2020b).

Although the above studies have explored the application of engineered EVs in the field of IDD, there is still space for further exploration in the innovation of engineering methods and EV action pathways and mechanisms. Of course, this also requires further exploration of IDD mechanisms and related research and application of biomaterials.

### 5.4 Other DOD

Because of the low incidence rate and the characteristics of the disease models, compared with OA, OP, and IDD, there are few studies on engineered EVs treatment of other degenerative bone diseases. At present, there is no research on engineered EVs for the treatment of osteonecrosis or degenerative tendinopathy. But a previous study has reported that the delivery of recombinant human bone morphogenetic protein 2 (rhBMP-2) using hydrogel can increase bone formation when used to treat drug-related jaw necrosis (MRONJ) (Brierly et al., 2019). Another study has shown that platelet-derived EV increases the expression of tendon markers, promotes remodeling of healthy extracellular matrix (ECM) and synthesis of anti-inflammatory mediators, and promotes the recovery of the human tendon-derived cell (hTDC) phenotype from a diseased state to a healthy state (Graça et al., 2022). The engineered EVs are likely to become a potential new treatment strategy for Osteonecrosis and degenerative tendinopathy in the future. Engineered EVs targeting diseased tissues can be used as a highly effective drug delivery tool with good biocompatibility and play an important role in tendinopathy and degenerative tendinopathy.

## 6 Conclusion, current challenges, and outlook

As a new cell-free strategy, extracellular vesicles (EVs) have demonstrated excellent anti-inflammatory, immunomodulatory, growth support, and drug delivery capabilities, and have demonstrated excellent performance in recent years in the field of degenerative orthopedic diseases. Engineered strategies based on cargo loading, surface modification, and artificial synthesis give EV greater biocompatibility, tissue targeting, therapeutic efficacy, and safety.

A typical feature of degenerative orthopedic diseases is that over time, the disease becomes more and more serious, usually accompanied by inflammation, cell destruction and death, ECM disorder and other pathological changes. Therefore, in addition to targeting specific signal pathways and pathogenesis, targeted treatment of EV is also very important.

Compared with tumors, cardiovascular diseases, pulmonary diseases and other diseases that have been studied more in the field of engineered EV, degenerative orthopedic diseases are limited by the disease model itself. Except for OP, the treatment method of intravenous injection of EV cannot be used, and only local injection of EV can be used. Therefore, the EV engineering strategy designed for OA and IDD mainly focuses on enhancing the effectiveness of EV itself and better mitigation strategies. In the study of OP, the main purpose is to improve the bone tissue targeting of EV. In addition, it is difficult to use EV as a biomarker to judge the degree and prognosis of degenerative orthopedic diseases. Therefore, in the field of engineered EVs for degenerative orthopedic diseases, the research prospects and innovation space are mainly focused on (Atesok et al., 2017): Modifying the EV membrane to enhance the tissue targeting and biocompatibility of EV, which depends on the relevant research of cell biology and biomaterials (Pei et al., 2022); Replacing EV cargo to enhance the biological activity and therapeutic effect of EV depends on basic research on disease related mechanisms (Calvit and Aranda, 1995); To improve the delivery mode of EV to adapt to different disease models (Wu et al., 2022); Biomimetic artificial nanoparticles with specific functions are designed based on material science methods (Xie et al., 2020); To further promote the clinical application of engineered EV in the field of degenerative orthopedic diseases.

Although more and more EV engineering methods have been designed, we should pay attention to their adaptability to the disease model itself and avoid the overuse of EV engineering methods in degenerative orthopedic diseases. We should consider whether the actual utility of engineered EVs is worth the high cost of complex engineering methods, and pay attention to its feasibility, safety and effectiveness in clinical model application (Yang et al., 2019; Yan et al., 2021b).
